# Chemerin-156 is the Active Isoform in Human Hepatic Stellate Cells

**DOI:** 10.3390/ijms21207555

**Published:** 2020-10-13

**Authors:** Marlen Spirk, Sebastian Zimny, Maximilian Neumann, Nichole McMullen, Christopher J. Sinal, Christa Buechler

**Affiliations:** 1 Department of Internal Medicine I, Regensburg University Hospital, 93053 Regensburg, Germany; marlen.spirk@stud.uni-regensburg.de (M.S.); sebastian.zimny@med.uni-muenchen.de (S.Z.); mneumann@caritasstjosef.de (M.N.); 2 Department of Pharmacology, Dalhousie University, Halifax, NS B3H 4R2, Canada; Nichole.McMullen@Dal.Ca (N.M.); Christopher.Sinal@Dal.Ca (C.J.S.)

**Keywords:** Galectin-3, IL-6, Proliferation, Tango Assay, CMKLR1

## Abstract

The chemokine chemerin exists as C-terminally processed isoforms whose biological functions are mostly unknown. A highly active human chemerin variant (huChem-157) was protective in experimental hepatocellular carcinoma (HCC) models. Hepatic stellate cells (HSCs) are central mediators of hepatic fibrogenesis and carcinogenesis and express the chemerin receptors chemokine-like receptor 1 (CMKLR1) and G protein-coupled receptor 1 (GPR1). Here we aimed to analyse the effect of chemerin isoforms on the viability, proliferation and secretome of the human HSC cell line LX-2. Therefore, huChem-157, 156 and 155 were over-expressed in LX-2 cells, which have low endogenous chemerin levels. HuChem-157 produced in LX-2 cells activated CMKLR1 and GPR1, and huChem-156 modestly induced GPR1 signaling. HuChem-155 is an inactive chemerin variant. Chemerin isoforms had no effect on cell viability and proliferation. Cellular expression of the fibrotic proteins galectin-3 and alpha-smooth muscle actin was not regulated by any chemerin isoform. HuChem-156 increased IL-6, IL-8 and galectin-3 in cell media. HuChem-157 was ineffective, and accordingly, did not enhance levels of these proteins in media of primary human hepatic stellate cells when added exogenously. These analyses provide evidence that huChem-156 is the biologic active chemerin variant in hepatic stellate cells and acts as a pro-inflammatory factor.

## 1. Introduction

Chemerin is a multifunctional protein with high expression in adipocytes and hepatocytes [1]. Chemokine-like receptor 1 (CMKLR1) and G Protein-Coupled Receptor 1 (GPR1) are functional chemerin receptors [2,3]. CMKLR1 is expressed by various tissues and cells including cells of the innate and adaptive immune system [4]. According to the Human Protein Atlas, GPR1 mRNA was hardly detectable in immune cells [5] and a separate study could not identify GPR1 mRNA in macrophages [6]. In murine peritoneal exudate cells, GPR1 mRNA was 0.02 to 0.03% of CMKLR1 mRNA levels [7]. In adipose tissues, GPR1 mRNA was highly abundant in the stromal vascular cell fraction, which is composed of fibroblasts, mesenchymal stem cells, endothelial cells, as well as smooth muscle cells and macrophages [8,9]. Chemerin is a well described chemoattractant and can act via both receptors to stimulate chemotaxis of immune or non-immune cells [1,10,11,12].

Immune cells in the tumor microenvironment can promote or suppress cancer growth [13,14]. In melanoma chemerin increased the ratio of anti-cancerous to pro-cancerous immune cells and elicited an anti-tumor response [15]. The chemerin induced shift from a tumor-promoting to a tumor-suppressive immune environment further protected from hepatocellular carcinoma (HCC). Here, chemerin lowered the release of IL-6 from tumor-adjacent endothelial cells and granulocyte-macrophage colony-stimulating factor from tumor cells [16]. Moreover, chemerin reduced HCC cell migration and invasion by weakening of the CMKLR1—phosphatase and tensin homolog (PTEN) complex [17].

Human chemerin is secreted as an inactive 163-amino acid protein (prochemerin). C-terminal processing produces 156, 157 and 158-amino acid proteins, all of which are biologically active. Further proteolysis generally results in the production of chemerin isoforms with less or absent biological activity. HuChem-157 (or its murine homolog Chem-156) was most effective in chemotaxis assays and thus, was used in nearly all in-vitro and in-vivo studies so far [1,10].

The main chemerin isoform in human plasma is huChem-163 [18]. While obesity is associated with increased systemic levels of chemerin, this was not associated with higher chemerin activity [18,19]. In contrast, the fraction of processed, active chemerin was reported to be increased in inflammatory conditions such as arthritis synovial fluids and ascites [20]. These findings suggest that inflammatory processes induce proteolytic cleavage and activation of prochemerin. Thus, studies of the function and distribution of chemerin isoforms in different body fluids, cells and tissues are important to clarify the multifaceted roles of chemerin. A further approach is to determine the activation of CMKLR1 and/or GPR1 signaling by chemerin contained in body fluids or cell supernatants [1,10]. Most studies used the Tango assay to analyse chemerin induced recruitment of beta-arrestin 2 to CMKLR1 or GPR1 [1,19,21]. These receptors also recruit beta-arrestin-1, and corresponding signaling was less well investigated [2].

Myofibroblasts are central players in the pathogenesis of fibrosis and tumors [22,23,24]. These cells express alpha-smooth muscle actin (α-SMA) and secrete extracellular matrix proteins, which are mostly collagens [22,23,24]. Moreover, these cells release cytokines (IL-6) and chemokines (IL-8, CCL2, CCL5) to regulate immune cell function, angiogenesis, and tissue fibrosis [24]. Myofibroblast secreted chemerin attracted bone-marrow derived mesenchymal stem cells in esophageal squamous cancer indicating a function in tumor progression [25]. In gastric cancer, myofibroblast released chemerin increased the migration and invasion of gastric carcinoma cells. Both chemerin receptors mediated the effects of chemerin on gastric cancer cells [11]. Myofibroblasts express CMKLR1 and GPR1 and autocrine/paracrine effects of chemerin may contribute to cancer pathogenesis [11].

Liver myofibroblasts are derived from resident hepatic stellate cells (HSC). HSCs are central players in fibrotic liver diseases and HCC [23,24]. Primary human HSCs express CMKLR1 and may very well respond to chemerin [26]. HSCs also secrete chemerin enabling autocrine/paracrine signaling pathways [27]. In the current investigation, the effects of different chemerin isoforms were studied in the human HSC cell line LX-2, which retains key features of human HSCs [28]. Overexpression of huChem-156, but not huChem-157, increased galectin-3, IL-6 and IL-8 in cell media. We therefore hypothesized that huChem-157 is not active in these cells. Recombinant huChem-157 had no effect on the levels of galectin-3, IL-6 and IL-8 in cell media of primary human HSCs providing further evidence for this suggestion.

## 2. Results

### 2.1. Overexpression of Chemerin Isoforms in LX-2 Cells

**Figure 1:**
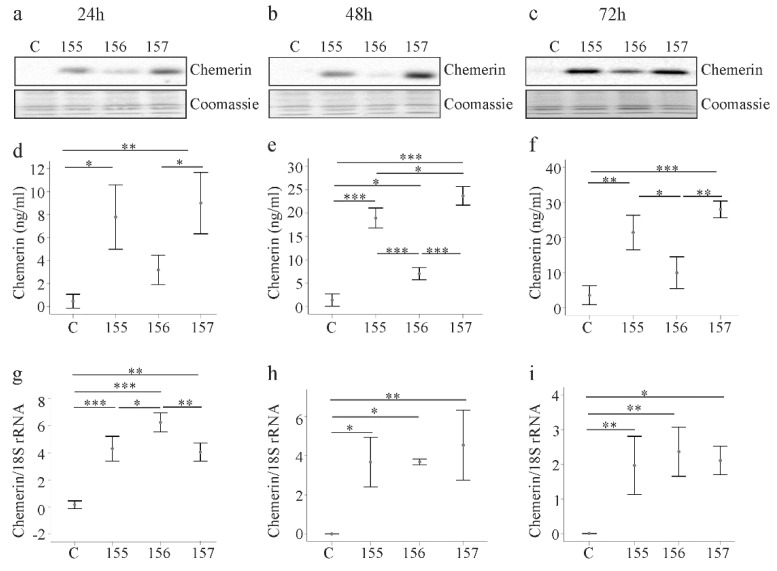
Expression of chemerin isoforms in LX-2 cells. (**a**–**c**) LX-2 cells were transfected with an insertless plasmid (C) or plasmids to express huChem-155, 156 or 157. Cellular chemerin protein was analyzed by immunoblot at 24, 48 and 72 h post-transfection. Coomassie stained membrane served as control; (**d**–**f**) Soluble chemerin protein was analyzed by ELISA at 24, 48 and 72 h post-transfection; (**g**–**i**) Chemerin mRNA was analyzed by real-time PCR at 24, 48 and 72 h post-transfection. N = 3. * *p* < 0.05, ** *p* < 0.01, *** *p* < 0.001.

LX-2 cells were transfected with the recombinant plasmids to overexpress chemerin variants. A chemerin antibody, which reacts with all isoforms, was used for immunoblot analysis. Chemerin was barely detectable in cell lysates of LX-2 cells transfected with the insertless vector (Figure 1a). In LX-2 cells transfected with the recombinant plasmids, huChem-155 and huChem-157 proteins were similarly abundant, while huChem-156 was less strongly expressed (Figure 1a). The observation that huChem-155 was well recognized by the chemerin antibody suggests that the apparent lower abundance of huChem-156 protein was not a technical issue related to the ability of the antibody to detect different forms of chemerin. The recombinant chemerin isoforms were detected at 24, 48 and 72 h post-transfection and protein expression patterns were comparable at these three time points (Figure 1a–c).

Soluble chemerin was measured with a pan-chemerin ELISA in the respective LX-2 supernatants. Chemerin levels were elevated in the supernatant of cells transfected with the recombinant vectors 24, 48 and 72 h post-transfection (Figure 1d–f). These levels were significantly higher than control for all isoforms at all time points with the exception of huChem-156 24 h and 72 h post-transfection (Figure 1d–f). The concentrations of chemerin in the cell supernatants increased over time regardless of the isoform type and were about 3-fold higher at 72 h in comparison to levels at 24 h after transfection (Figure 1d–f).

Consistent with these results, chemerin mRNA levels were also increased in LX-2 cells transfected with the recombinant plasmids (Figure 1g–i). Expression of chemerin mRNA was highest in huChem-156 producing LX-2 cells at 24 h post-transfection. At subsequent time points, chemerin mRNA levels were comparable in the LX-2 cells transfected with the recombinant plasmids, and were significantly higher than in the control transfected cells (Figure 1g–i).

While we cannot rule out differential antigenicity of the individual chemerin forms with respect to the detection antibodies used in these analyses, the apparent disparity in the relative protein and mRNA levels for the transfected cells suggests that posttranscriptional and/or posttranslational mechanisms contribute to lower cellular and soluble huChem-156 protein (Figure 1a–f).

### 2.2. Analysis of Chemerin Isoform Activity and Chemerin Receptor Expression

**Figure 2:**
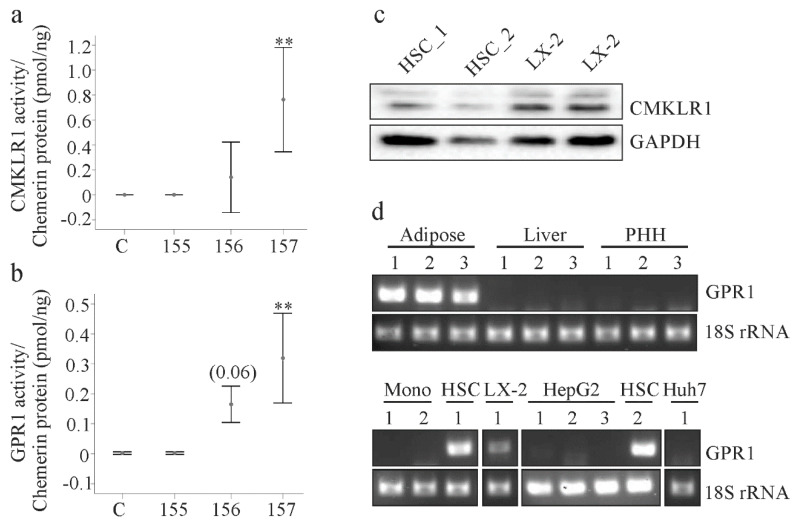
Chemerin activity and expression of chemerin receptors. (**a**) Activation of CMKLR1 by chemerin isoforms in the supernatants of the transfected LX-2 cells. Activity relative to chemerin concentration is shown (*n* = 4); (**b**) Activation of GPR1 by chemerin isoforms in the supernatants of the transfected LX-2 cells. Activity relative to chemerin concentration is shown. Number in brackets is the *p*-value (*n* = 4); (**c**) CMKLR1 protein in LX-2 cells and primary human hepatic stellate cells (HSC_1, HSC_2 of 2 donors); (**d**) GPR1 mRNA in human adipose tissues of three donors, in liver tissues of three donors, in primary human hepatocytes (PHH) of three donors, in primary human monocytes (Mono) of two donors, in HSC of two donors and in the cell lines LX-2, HepG2 and Huh7. ** *p* < 0.01.

Chemerin receptor activation was measured using supernatants of LX-2 cells 24 h post-transfection. Both CMKLR1 and GPR1 were activated by cell culture medium of huChem-157 overexpressing cells (Figure 2a,b). In contrast, huChem-156 did not activate CMKLR1 and only modestly activated GPR1 (*p* = 0.06; Figure 2a,b). HuChem-155 was inactive with respect to either receptor (Figure 2a,b).

Previous work by our group showed that CMKLR1 protein was expressed by primary human hepatic stellate cells (HSCs) [26]. Consistent with this, CMKLR1 protein was readily detected in HSC and LX-2 cells in the current study (Figure 2c). Reverse transcription-PCR revealed that GPR1 mRNA was present in human adipose tissues, but not in human liver or primary human hepatocytes (Figure 2d). Accordingly, GPR1 mRNA was not detectable in the human hepatocyte cell lines HepG2 and Huh7, nor was it detected in human monocytes (Figure 2d). In contrast GPR1 mRNA was readily detected in LX-2 and HSC cells (Figure 2d).

Two different GPR1 antibodies were employed to determine GPR1 protein levels. Both detected GPR1 in HepG2 and Huh7 cells and in primary human hepatocytes indicating potential problems with the specificities of the antibodies (Supplementary Figure S1).

### 2.3. Effect of Chemerin Isoforms on Proliferation and Cytotoxicity in LX-2 Cells

**Figure 3:**
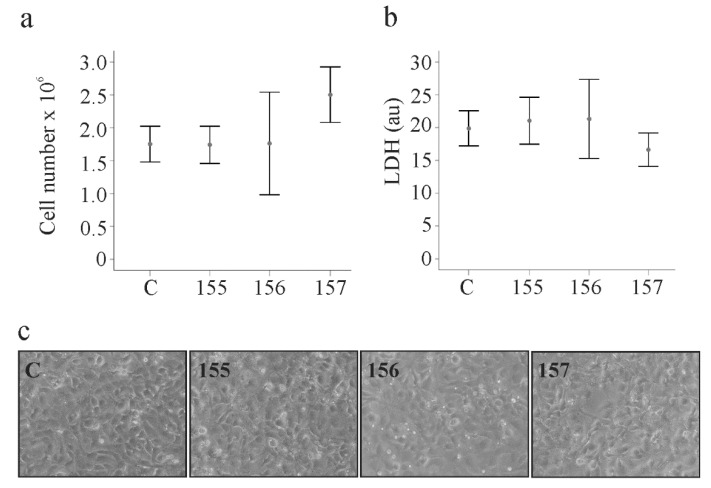
Chemerin isoform effects on cell proliferation, lactate dehydrogenase and cell morphology. (**a**) LX-2 cells were transfected with an insertless plasmid (C) and plasmids to express huChem-155, 156 and 157. Cell number was counted at 48 h post-transfection (*n* = 3); (**b**) Lactate dehydrogenase (LDH) in the supernatants of the cells described in a (*n* = 4). (**c**) Morphology of the transfected cells.

Proliferation of HSCs is a characteristic of activated cells and contributes to liver diseases [22]. Cell numbers were counted 24, 48 and 72 h post-transfection and growth rates were not affected by the chemerin variants (Figure 3a and Supplementary Table S1). Soluble levels of lactate dehydrogenase as a measure of cytotoxicity were comparable between the cells transfected with the different plasmids at each time point (Figure 3b and Supplementary Table S2). Overexpression of the chemerin isoforms did not affect cell morphology, which was investigated by light microscopy (Figure 3c).

### 2.4. Effect of Chemerin Isoforms on Alpha-Smooth Muscle Actin and Galectin-3

**Figure 4:**
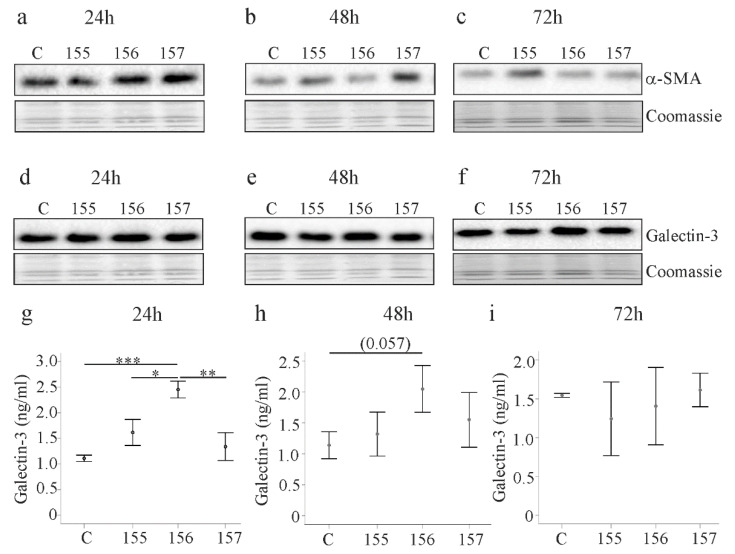
Alpha-smooth muscle actin (α-SMA) and galectin-3 in LX-2 cells expressing chemerin isoforms. (**a**–**c**) LX-2 cells were transfected with an insertless plasmid (C) and plasmids to express huChem-155, 156 and 157. Cellular α-SMA was analyzed by immunoblot at 24, 48 and 72 h post-transfection. Coomassie stained membrane served as control; (**d**–**f**) Galectin-3 in the cells described in a–c; (**g**–**i**) Galectin-3 in the cell media of LX-2 cells described in a–c. N = 3; * *p* < 0.05, ** *p* < 0.01, *** *p* < 0.001, the number in (**h**) in brackets is the *p*-value.

Alpha-smooth muscle actin (α-SMA) is a marker of activated HSCs [22,28]. Expression of the different chemerin isoforms did not change α-SMA protein 24, 48 and 72 h post-transfection (Figure 4a–c). Galectin-3 is a key factor in organ fibrosis [29] and, despite unchanged cellular levels, galectin-3 was induced in media of LX-2 cells expressing huChem-156 24 h post-transfection (Figure 4d,g). This effect persisted for up to 48 h and disappeared at 72 h (Figure 4h,i).

### 2.5. Effect of Chemerin Isoforms on IL-6, IL-8 and Pentraxin 3

**Figure 5:**
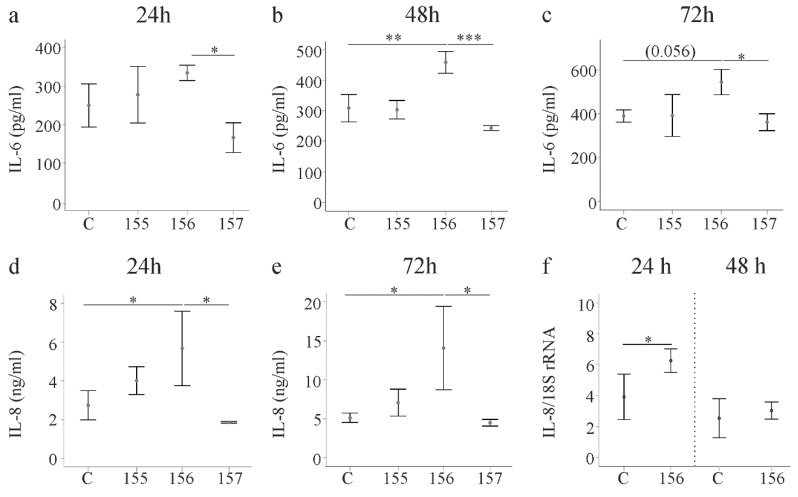
IL-6 and IL-8 in media of LX-2 cells expressing chemerin isoforms. (**a**–**c**) LX-2 cells were transfected with an insertless plasmid (C) and plasmids to express huChem-155, 156 and 157. IL-6 was analyzed by ELISA at 24, 48 and 72 h post-transfection; (**d**,**e**) LX-2 cells were transfected with an insertless plasmid (C) and plasmids to express huChem-155, 156 and 157. IL-8 was analyzed by ELISA at 24 and 72 h post-transfection; (**f**) IL-8 mRNA in these cells at 24 and 48 h post-transfection. * *p* < 0.05, ** *p* < 0.01, *** p < 0.001the number in c in the bracket is the *p*-value. N = 3 for all data shown.

Activated HSCs secrete various cytokines and chemokines. IL-6 was induced by huChem-156 at all of the time points and this effect was significant at 48 h post-transfection. Of note, huChem-156 expressing LX-2 cells had higher IL-6 than huChem-157 producing cells at all time points (Figure 5a–c). IL-8 recruits neutrophils and thereby contributes to liver disease [30]. IL-8 was higher in huChem-156 expressing cells in comparison to the controls and huChem-157 producing LX-2 cells (Figure 5d,e and data not shown). This up-regulation was significant 24, 48 and 72 h after transfection (Figure 5d,e and Supplementary Figure S2). An induction of IL-8 mRNA occurred at 24 h post-transfection (Figure 5f).

Pentraxin 3 is produced by activated HSCs and modulates cytokine and chemokine production [31]. None of the chemerin isoforms affected pentraxin 3 levels in the cell supernatants, which was measured 24, 48 and 72 h after transfection (Supplementary Table S3).

### 2.6. Secretome of Primary Human HSCs and LX-2 cells

**Figure 6:**
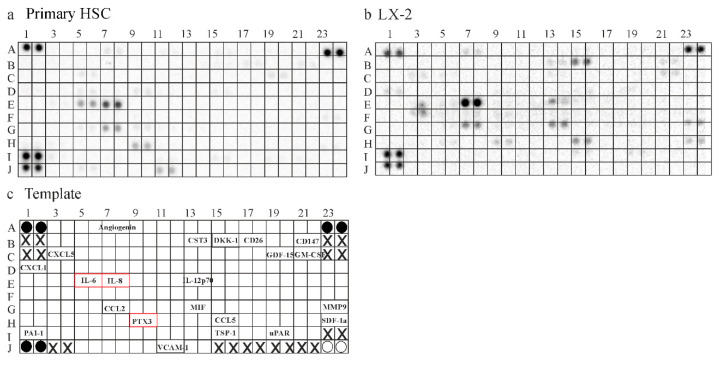
Secretome of HSC and LX-2 cells. (**a**) Hybridization of the human Cytokine Array with supernatant of primary human HSC; (**b**) Hybridization of the human Cytokine Array with supernatant of LX-2 cells; (**c**) Template of the Array: Black circles are the reference spots. White circles are negative controls and X marks positions where no antibodies were spotted. The names of the soluble factors detected by either array are given at the respective position of the Cytokine Array. Proteins, which were further analyzed by ELISA, are in red boxes.

Contrary to expectations, huChem-157, which is the most active chemerin isoform [1,10], did not affect IL-6, IL-8 or galectin-3 in LX-2 cell media. To further investigate this finding, experiments with primary human HSCs were performed. Firstly, the secretome of LX-2 cells and primary HSCs was compared by the use of a human Cytokine Array, which contained 105 different capture antibodies (Figure 6a–c). This experiment indicated that LX-2 cells produced a greater variety of these soluble molecules. Interestingly, IL-6 could only be detected in supernatants of the primary HSCs, which also had increased levels of pentraxin 3. IL-8 may be higher in supernatants of the LX-2 cell line. While this screening experiment produced interesting preliminary results, confirmatory analysis was needed to prove a possible differential abundance of further soluble factors between HSCs and LX-2 cells (Figure 6a,b).

**Figure 7:**
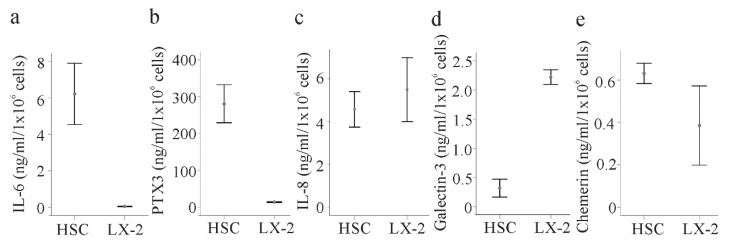
Secretome of HSCs and LX-2 cells. (**a**) IL-6 in the supernatant of HSCs of three different donors and LX-2 cells; (**b**) Pentraxin 3 (PTX3); (**c**) IL-8; (**d**) galectin-3; and (**e**) chemerin in the cell culture media of these cells.

To provide evidence for a differential abundance of some soluble molecules between HSCs and LX-2 cells, these proteins were measured in the media of LX-2 cells and primary HSCs isolated from the liver of three different patients by ELISAs. IL-6 and pentraxin 3 were indeed about 6-fold and 30-fold higher in the primary cells, respectively (Figure 7a,b). IL-8 was nevertheless similar in both cell types (Figure 7c). Galectin-3 (not included in the Cytokine Array) was about 8-fold increased in the supernatants of LX-2 cells compared to the primary HSCs (Figure 7d). Chemerin may be higher in the primary cell supernatant (Figure 7e).

### 2.7. Effect of huChem-157 on IL-6, IL-8, Pentraxin 3 and Galectin-3 in Primary Human HSCs

**Figure 8:**
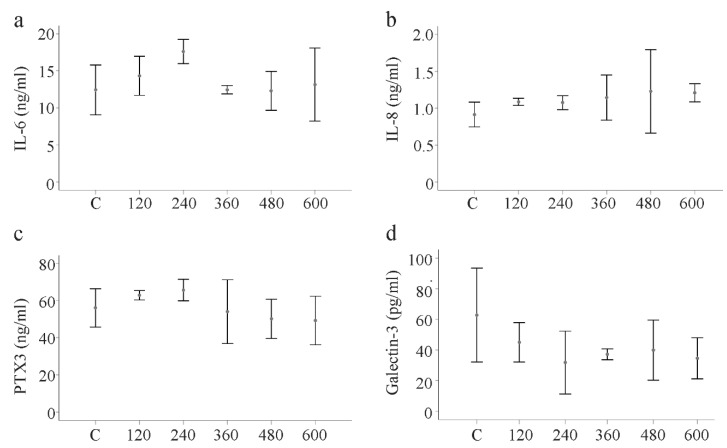
Effect of huChem-157 on IL-6, IL-8, pentraxin 3 (PTX3) and galectin-3 in primary human HSCs of three donors. (**a**) HSCs were incubated with increasing concentrations of recombinant huChem-157 for 24 h and IL-6; (**b**) IL-8; (**c**) PTX3 and (**d**) galectin-3 were measured by ELISA.

The primary HSCs of three donors were incubated in medium supplemented with recombinant huChem-157 (120, 240, 360, 480 and 600 ng/mL) for 24 h. Analysis of IL-6, IL-8, pentraxin 3 and galectin-3 in the supernatants showed that none of these soluble mediators was impacted by the huChem-157 (Figure 8a–d).

## 3. Discussion

The results from the present study provide evidence that huChem-156 up-regulates the cytokine IL-6, the chemokine IL-8 and the fibrotic protein galectin-3 in the human hepatic stellate cell line LX-2. In contrast, huChem-157, which is generally regarded as the most active isoform [1,10], was ineffective in the cell line. Moreover, recombinant huChem-157 could not induce these soluble proteins in primary human HSCs.

Myofibroblasts are central players in organ fibrosis and chemerin released by these cells functions as a chemoattractant for tumor cells [12,25]. The autocrine/paracrine effects of chemerin on myofibroblasts are less well understood. Hepatic stellate cells express both functional chemerin receptors. This was shown for CMKLR1 in a previous study [26] and for GPR1 in the present analysis although only mRNA expression has been analyzed to date. Thus, these cells are likely responsive to chemerin. It should be noted that GPR1 mRNA was detected in LX-2 cells, primary HSCs and adipose tissues, the latter of which served as a positive control. Primary human hepatocytes and monocytes did not express GPR1 mRNA. These findings are in agreement with previous reports showing expression of GPR1 in fat tissues and myofibroblasts and a lack of GPR1 in monocytes [6,7,9]. CMKLR1 is expressed on hepatocytes and hepatic Kupffer cells, which represent liver resident macrophages [26]. The physiological and pathophysiological relevance of the more restricted expression of GPR1 in liver cells needs further research.

The LX-2 cell line retains key features of primary HSCs. Global gene expression analysis revealed a 98.7% similarity in gene expression between LX-2 cells and primary HSCs [28]. Herein we showed that LX-2 cells have lower levels of soluble IL-6, pentraxin 3 and possibly chemerin and greatly elevated concentrations of galectin-3 than the primary HSCs. IL-8 was comparable between both cell types. Considering the key function of galectin-3, IL-6, chemerin and pentraxin-3 in inflammation and cancer [16,29,31,32,33], the altered secretome may be a consequence of malignant cell transformation. This hypothesis needs to be tested in future studies.

The primary goal of the present work was to clarify the effects of chemerin in HSCs. Therefore, the active isoforms huChem-157 and huChem-156 and the inactive isoform huChem-155 were overexpressed in LX-2 cells. While protein levels of huChem-156 were the lowest of all isoforms, analysis of mRNA levels revealed comparable expression of all recombinant isoforms. This suggests that posttranscriptional and/or posttranslational mechanisms may contribute to reduced protein levels. Cells secrete prochemerin and C-terminal processing occurs extracellularly [1,10], and thus, this observation may have limited physiological relevance.

The Tango assay determines beta-arrestin 2 recruitment upon addition of a suitable ligand [1,34]. As expected, recombinant huChem-157 was most effective in the CMKLR1 and GPR1 Tango assays. HuChem-156 only activated GPR1, and huChem-155 was ineffective in both assays. Interestingly, soluble chemerin levels increased over time excluding a role of LX-2 cells in chemerin elimination or excessive degradation.

None of the chemerin variants was cytotoxic and cell proliferation was normal. Characteristic proteins produced by these cells are α-SMA and galectin-3, and their cellular levels did not change upon chemerin over-expression. Soluble galectin-3 was nevertheless about 2-fold higher in huChem-156 producing cells. This effect was significant at 24 h after transfection and completely disappeared by 72 h. IL-8 was also elevated in the supernatant of huChem-156 expressing cells and this effect persisted over time. IL-6 was higher at all time points with a significant effect at 48 h post-transfection. None of these soluble factors were regulated by huChem-157. Analysis of IL-8 mRNA levels showed that higher IL-8 protein was paralleled by higher IL-8 mRNA levels in LX-2 cells expressing huChem-156. Recent studies described a role of murine Chem-156, corresponding to huChem-157, in the activation of nuclear factor kappa B and subsequent release of inflammatory proteins [16,35]. This pathway may also be involved in the pro-inflammatory effects of huChem-156.

CMKLR1 was not activated by huChem-156 and at least the CMKLR1-beta-arrestin 2 pathway may not play a role herein. HuChem-156 modestly increased the GPR1 induced recruitment of beta-arrestin 2. GPR1 responded to huChem-157 and 156, but only the latter isoform had an effect in the LX-2 cells. This may argue against a role of the GPR1—beta-arrestin 2 pathway in the up-regulation of the inflammatory metabolites. However, chemerin concentration and exposure time are relevant for the effects of chemerin. Short-term exposure to chemerin increased levels of phosphorylated Akt, which declined below basal levels upon longer time stimulation [17]. Low, but not high, chemerin concentrations induced ERK1/2 and p38 MAPK phosphorylation [36]. Recruitment of beta-arrestins to GPR1 and CMKLR1 nevertheless increased with higher chemerin concentrations [2]. Accordingly, lower activation of GPR1 by huChem-156 compared to huChem-157 may be linked to an increased expression of inflammatory factors and this may not occur by the more active ligand. Whether GPR1—beta-arrestin 2 signaling contributes to higher production of the soluble factors described above has not yet been evaluated.

Pentraxin-3 and α-SMA are markers of activated HSCs [31,37,38] and were not regulated by any of the chemerin isoforms. HuChem-157 had no effect on stellate cell proliferation, activation and release of pro-inflammatory factors, all of which contribute to liver injury [22,30,38]. Accordingly, diethylnitrosamine induced liver fibrosis was not changed in mice with hepatic overexpression of the murine homolog of huChem-157, Chem-156 [39].

HuChem-156 is produced by cathepsin G, chymase or kallikrein 7 from huChem-163 [1]. These proteases are expressed by mast cells, which contribute to liver fibrosis and HCC [40,41,42]. Whether huChem-156 is abundant in human liver needs further analysis. Studies in mice identified murine Chem-155 (the mouse homolog of huChem-156) in liver tumors [39], and in terms of the present results, this isoform may contribute to a pro-inflammatory state in the cancer tissues.

## 4. Materials and Methods

### 4.1. Primary Human Cells and Cell Lines

Cell suspensions depleted in primary human hepatocytes and enriched in non-parenchymal cells were obtained from Hepacult (Regensburg, Germany) and used for the isolation of hepatic stellate cells. The cell suspension was centrifuged at 50 g for 5 min to recover the non-parenchymal cells. Cells were resuspended in 10 mL DMEM/high-glucose medium (supplemented with 10% fetal bovine serum and 1% penicillin/streptomycin) and transferred to cell culture flasks. The medium was changed 90 min later, and then every day for 4 days. Two weeks later, the flasks contained almost exclusively human hepatic stellate cells. These cells were stimulated with recombinant huChem-157 (R&D Systems, Wiesbaden-Nordenstadt, Germany) in serum-free medium.

The LX-2 human hepatic stellate cell line was obtained from Merck Chemicals GmbH (Darmstadt, Germany) and cells were cultivated in DMEM medium with 2% fetal bovine serum.

Lactate dehydrogenase (LDH) in the cell supernatants was measured by the Cytotoxicity Detection Kit from Roche (Mannheim, Germany). Cells were counted by the Countess II FL from Life Technologies (Thermo Fisher Scientific, Waltham, MA, USA). This approach discriminates live and dead cells.

### 4.2. Expression of Recombinant Human Chemerin Isoforms in LX-2 Cells

Polymerase chain reaction (PCR) to obtain human chemerin cDNA was done with the universe primer 5′- CGA AAG CTT ATG CGA CGG CTG CTG ATC C -3′ and the reverse primers huChem-157: 5′- CGA CCG CGG TTA GGA GAA GGC GAA CTG TCC AGG -3′, huChem-156: 5′- CGA CCG CGG TTA GAA GGC GAA CTG TCC AGG GAA-3′ and 5′- huChem-155 CGA CCG CGG TTA GGC GAA CTG TCC AGG GAA GTA-3′. The cutting sites for the restriction enzymes HindIII and SacII are underlined. The DNA was cloned in the vector pcDNA3.1 (Thermo Fisher Scientific, Waltham, MA, USA). The DNA sequences of the fragments were verified by sequence analysis (GeneArt, Regensburg, Germany). Transfection of cells was done with Lipofectamine^TM^3000 Reagent (Thermo Fisher Scientific).

### 4.3. Monitoring of Gene Expression by Real-Time RT-PCR

The RNeasy Mini Kit was from Qiagen (Hilden, Germany) and oligonucleotides from Metabion (Planegg-Martinsried, Germany). LightCycler FastStart DNA Master SYBR Green I was from Roche (Mannheim, Germany). Gene expression was analyzed by semiquantitative real-time PCR. Total cellular RNA was isolated and 1 µg RNA was reverse transcribed (Promega Reverse Transcription System, Promega, Madison, WI, USA) in a volume of 40 µL; 2 µL of the cDNA was used for amplification in glass capillaries. Human GPR1 was amplified with the primers 5´-AGC CAC AGG CAC CGG CAA-3′ and 5´-TCC AAA TCA GAC TCC AGA GAG- 3′. Human chemerin was amplified with the primers 5´-CAG GAG ACC AGT GTG GAG A-3´and 5-GTG AGG ACC CCC ACA GCT-3´ and 18S rRNA with 5′-GAT TGA TAG CTC TTT CTC GAT TCC-3′ and 5′-CAT CTA AGG GCA TCA CAG ACC-3′. The primers 5´-ACC GGA AGG AAC CAT CTC ACT GT-3´and 5´-GCA TCT GGC AAC CCT ACA ACA-3´ were used to amplify human IL-8 mRNA. The specificities of the PCRs were confirmed by sequencing of the amplified DNA fragments (Geneart, Regensburg, Germany). The second derivative maximum method was used for quantification with the LightCycler software.

### 4.4. SDS-Polyacrylamide Gel Electrophoresis and Immunoblotting

Proteins (20 µg) were separated by SDS-polyacrylamide gel electrophoresis and blotted to polyvinylidene fluoride membranes (Bio-Rad, Munich, Germany). Membranes were incubated with the antibodies in 1.5% BSA/PBS/0.1% Tween and detection of the immune complexes was performed with the ECL Western blot detection system (Amersham Pharmacia, Deisenhofen, Germany). The antibodies used were obtained from the following sources: human chemerin, R&D Systems (Wiesbaden-Nordenstadt, Germany); GAPDH, New England Biolabs GmbH (Frankfurt am Main, Germany); galectin-3, BD Biosciences (Heidelberg, Germany); α-SMA and CMKLR1, Abcam (Cambridge, UK); GPR1, Bioss (Woburn, MA, USA) and Abnova (Taipei City, Taiwan). ImageJ software was used for quantification [43].

### 4.5. ELISAs and Cytokine Array

ELISAs were ordered from R&D Systems and performed as recommended by the distributor. Proteom Profiler^TM^ Human XL Cytokine Array was from R&D Systems and was hybridized with cell culture media as recommended by the company.

### 4.6. Tango Assay

Chemerin activation of CMKLR1 and GPR1 was determined by the Tango assay as described [19,21].

### 4.7. Statistical Analysis

Data are presented as mean ± standard deviation. Statistical differences were analyzed by ANOVA with post-hoc Tukey, Mann Whitney U-test (SPSS Statistics 25.0 program, IBM, Leibniz Rechenzentrum, München. Germany) or Student’s *t*-test (MS Excel), and a value of *p* < 0.05 was regarded significant.

## 5. Conclusions

The present study identified huChem-156 as the active chemerin isoform in human hepatic stellate cells. The physiological and pathophysiological role of this variant in liver fibrosis and HCC warrants further studies.

## Supplementary Materials

The following are available online at https://www.mdpi.com/1422-0067/21/20/7555/s1.
