# Supplementary material for: Chemerin-156 is the Active Isoform in Human Hepatic Stellate Cells
Source: Int J Mol Sci. 2020 Oct 13;21(20):7555. doi: 10.3390/ijms21207555 (PMC7589075; doi:10.3390/ijms21207555)
Supplement: Supplementary file 1 [file ijms-21-07555-s001.pdf]

Chemerin-156 is the Active Isoform in Human Hepatic Stellate Cells, Supplementary figures and tables.

Marlen Spirk [1], Sebastian Zimny [1], Maximilian Neumann [1], Nichole McMullen [2], Christopher J. Sinal [2] and Christa Buechler [1,*]

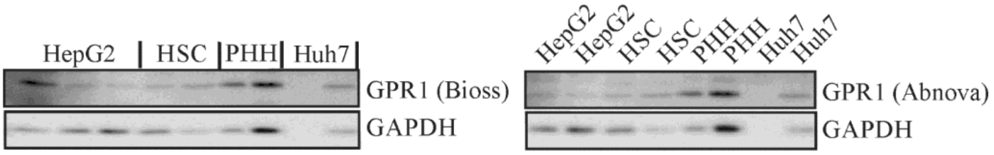

Supplementary Figure 1. Immunoblot analysis of GPR1 using the antibodies from Bioss and Abnova. Primary human hepatocytes (PHH), hepatic stellate cells (HSC).

Supplementary Table 1: Cell number (x10^{6}) at 24 and 72 h post-transfection. N = 3

	C	huChem-155	huChem-156	huChem-157
24h	1.8 ± 0.3	2.1 ± 1.2	2.2 ± 0.4	2.9 ± 2.0
72h	2.2 ± 0.5	2.6 ± 1.1	2.6 ± 0.7	3.2 ± 1.1

Supplementary Table 2: Analysis of lactate dehydrogenase (arbitrary units) at 24 and 72 h post-transfection. N = 4

	C	huChem-155	huChem-156	huChem-157
24h	16.8 ± 4.8	15.3 ± 5.9	15.8 ± 6.4	11.6 ± 6.7
72h	29.3 ± 6.2	28.9 ± 5.6	28.5 ± 6.5	25.1 ± 3.5

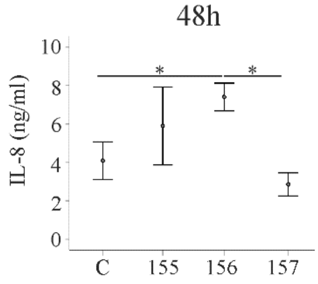

Supplementary Figure 2. IL-8 in media of LX-2 cells expressing chemerin isoforms 48 h post-transfection. *$p < 0.05$, N = 3.

Supplementary Table 3: Analysis of pentraxin 3 (ng/ml) 24, 48 and 72 h post-transfection. N = 3

	C	huChem-155	huChem-156	huChem-157
24h	6.9 ± 0.5	9.6 ± 2.9	7.6 ± 0.4	6.6 ± 0.3
48h	10.8 ± 2.0	10.9 ± 2.0	10.6 ± 2.4	10.0 ± 2.0
72h	11.9 ± 1.4	11.6 ± 2.4	11.2 ± 0.7	12.4 ± 1.4
